# Protection of BALB/c mice against pathogenic *Brucella abortus *and *Brucella melitensis* by vaccination with recombinant Omp16

**DOI:** 10.22038/ijbms.2019.36369.8665

**Published:** 2019-11

**Authors:** Hamed Alizadeh, Mehrooz Dezfulian, Mehdi Rahnema, Jalil Fallah, Davoud Esmaeili

**Affiliations:** 1 Department of Microbiology, Karaj Branch, Islamic Azad University, Karaj, Iran; 2 Department of Microbiology, Zanjan Branch, Islamic Azad University, Zanjan, Iran; 3 Lister Laboratory of Microbiology, Tehran, Iran; 4 Department of Microbiology and Applied Microbiology Research Center, Systems Biology and Poisonings Institute, Baqiyatallah University of Medical Sciences, Tehran, Iran; 5 Applied Virology Research Center, Baqiyatallah University of Medical Sciences, Tehran, Iran

**Keywords:** BALB/c mice, Brucella, Brucellosis, Outer membrane protein, Recombinant protein

## Abstract

**Objective(s):**

Prevention of the globally spread zoonotic infection, brucellosis which affects an extensive range of hosts is still challenging researchers. There are no approved vaccines for the prevention of human disease and those used for animal brucellosis have adverse properties, which limit their application. We investigated the immunological and protective effects of recombinant 16 kDa outer membrane protein of *Brucella abortus* (Omp16) which introduced a new candidate for brucellosis subunit vaccine.

**Materials and Methods:**

*Brucella* Omp16 gene was cloned in pET-23a and expressed in *Escherichia coli *BL21 (DE3). Recombinant Omp16 (rOmp16) was purified using nickel resin and confirmed by Western blot analysis. BALB/c mice were immunized with rOmp16, afterward, specific serum antibodies and cytokine responses were evaluated. Protection of immunized mice against pathogenic *B. abortus *544 and *B. melitensis* 16M was evaluated by the intraperitoneal bacterial challenge.

**Results:**

Sequencing results of the recombinant plasmid vector along with Western blotting confirmed the cloning procedure. Recognition of rOmp16 by specific IgG from serum samples of infected cases suggests the stimulation of immune response to this protein. Significant total serum IgG along with remarkable IgG1 and IgG2a response to the protein was recorded. A significant increase in IFN-γ, and IL-4 levels were observed from splenocyte cultures of immunized mice which were stimulated with rOmp16 suggesting the development of T-lymphocyte mediated immunity against the recombinant antigen.

**Conclusion:**

The intraperitoneal challenge with *B. abortus* 544 and *B. melitensis* 16M confirmed that rOmp16 is able to elicit efficient protective immune responses in the animal host.

## Introduction

*Brucella *is Gram-negative, facultative intracellular bacteria which cause brucellosis in both animals and humans (1, 2). The most important *Brucella* pathogens for human and animals are *Brucella abortus*, *Brucella melitensis* and *Brucella suis* (2-4). The animal infection causes abortion and reduces fertility in livestock, which leads to significant economic losses all over the world (4, 5). Human brucellosis is known to have nonspecific manifestations such as undulant fever, osteomyelitis, and arthritis (1, 6-8). 

*B. melitensis *Rev 1 and *B. abortus* S19 are live attenuated vaccine strains that are currently used in sheep and cattle, respectively. They were successful in disease eradication and control programs in some countries (3, 9). However, there are considerable troubles associated with the use of these vaccine strains. The most important problems are development of agglutinating antibodies in vaccinated animals which are indistinguishable from those elicited by natural infection (10, 11). Moreover, antibodies against *Brucella* LPS could cross-react with some Gram-negative bacteria (12, 13). The new brucellosis vaccines overcome to these problems and have some properties that’s great importance to veterinary medicine. One of the major aims of researches in brucellosis is the identification of *Brucella *antigens, eliciting immune responses, which might be useful for the development of subunit vaccines or diagnostic tests that avoid the disadvantages of currently used ones (13-15).

Outer membrane proteins (OMPs) of *Brucella *are considered by far as the immunogenic component of the bacterial cell wall (13, 16) and there are many reports which describe their protective effects in animal models (17-25). Omp16 is an outer membrane protein which included in all *Brucella* strains (26-28). In this report, we describe the full-length cloning, expression, and purification of Omp16 and assessment of immunological properties of this recombinant protein in BALB/c mouse model. 

## Materials and Methods

***Bacterial strains and plasmid vectors***

*B. abortus *544 and *B. melitensis* 16M were routinely cultured on Brucella agar and incubated in 37 ^°^C for 72 hr. *Escherichia coli* DH_5_*α* *and* *E. coli* BL21 (DE3) were used as prokaryotic hosts for cloning and expression respectively and were cultured using LB broth/agar Merk- Germany. 

pJET1.2 (Thermo Scientific) and pET-28a (+) (Novagen) were applied as cloning and expression plasmid vectors. 

***Cloning of Omp16***

A primer pair was designed to amplify the whole coding region of *Omp16* which were F5’-TTAGGATCCATGCGCCGTATCCAGTCGAT-3 and R5’- AACAAGCTTTTACCGTCCGGCCCCGTTG -3’ including restriction sequences of *Bam*HI and *Hind*III at the 5’-end respectively (underlined). Complete ORF or *Omp16* gene was amplified with PrimSTAR^®^ HS DNA polymerase (TaKaRa) and Blunt ended amplified product was cloned in pJET1.2 plasmid vector by clonJET*® PCR Cloning Kit* (Thermo Scientific). Recombinant pJET1.2-*Omp16* constructs were sequenced with pJET1.2 specific primers (Thermo Scientific) in an ABI 3730xl DNA Analyzer machine. Sequencing results were analyzed with Vector NTI^TM^ advanced 11.0 (Invitrogen). The *Omp16* was then sub cloned in pET-23a (+) vector between *Bam*HI and *Hind*III restriction sites (pET28-*Omp16*). Using T7 promoter/terminator universal primers, recombinant plasmids were confirmed by sequencing as described above. 

***Protein expression and purification***

pET28-*Omp16 *was transformed to *E. coli* BL21(DE3). Recombinant colonies were grown in LB medium containing kanamycin (30 µg/ml) at 37 ^°^C to reach an OD_620_. Subsequently_,_ IPTG at the final concentration of 1 mM was added to the growing culture, in order to expression of recombinant Omp16 (rOmp16). Samples of three hours of induction at 37 ^°^C were collected along with a non-induction sample as the control. Sample lysates were analyzed by 12.5% resolving polyacrylamide in mini gels (Bio-Rad) and followed by Coomassie Brilliant Blue G-250 staining (29). 

The cell pellet from two liters of 2-hour induced culture was collected. rOmp16 was purified according to the previously described hybrid procedure (29). Totally, the cell pellet was suspended and lysed thoroughly in buffer containing 8 M urea (Buffer B, pH 8.5, QIAGEN). Cell debris were removed by centrifugation and Ni-NTA resin (QIAGEN) was added to the clear lysate, then mixed by rotation for one hour. The resin was washed twice with buffer C (8 M urea, pH 6.3) and urea was removed by washing the resin with buffers containing decreasing urea concentrations (8, 6, 4, 2, 1, and 0 M, pH 8.5). The column was washed with native wash buffer (40 mM imidazole, pH 8.5), and 6-His-tagged rOmp16 was eluted with 350 mM imidazole. Imidazole was subsequently removed by dialysis against PBS (pH 7.5). Recombinant protein was analyzed by SDS-PAGE and quantified by spectrophotometer NanoDrop^®^ 2000 (Thermo Scientific, USA). After purification of rOMP16, in order to remove LPS, EtEraser™ HP Endotoxin Removal Kit (BioEndo, China) was used according to related guideline. 

***Western blot analysis***

Western blot analysis was performed according to previously described procedures (29). Briefly, purified rOmp16 was electro-transferred from unstained 12.5% polyacrylamide gel to polyvinylidene difluoride (PVDF) membrane in a semidry transfer apparatus (Trans-Blot^® ^SD, Bio-Rad, USA). The sheet was blocked by 2% bovine serum albumin dissolved in PBS (pH 7.5) for one hour at 37°C while rocking. The protein was probed by rabbit polyclonal anti-serum against *Brucella* outer membrane protein complex (prepared in our laboratory). HRP conjugated anti-rabbit IgG applied to the sheet as the secondary antibody. The sheet was finally developed with chemiluminescent substrate (ECL) under standard conditions and the appearance of fluorescent bands was immediately recorded on radiographic films. Purified rOmp16 were also subjected to Western blot analysis and probed by 1:3000 dilutions of 3 serum samples from brucellosis patients. These were treated with HRP-Anti-Human IgG and developed as described above.

***Mouse immune response analysis***

Six- to seven-week-old Female BALB/c mice were obtained from the Laboratory Animal Production Department at Pasteur Institute of Iran (Research & Production Complex, Alborz). Animal maintenance, handling, and all experiments were performed with strict accordance to institutional ethical guidelines and international protocols (30). Mice were immunized subcutaneously with rOmp16 formulated in Freund’s adjuvant in three dose with 10-day intervals. Negative control non-immunized mice group received phosphate buffered saline simultaneously. The two vaccine groups were considered which received a single intraperitoneal dose containing 10^4^ CFU of *B. abortus *S19 and *B. melitensis* Rev1 (25). 

Mice were bled from the retroocular vein at days 0, 10, 20 (prior to antigen administration), 30, 40 and 50. Serum samples were collected and stored at -80 ^°^C. All samples were investigated for specific total serum IgG (Sigma, USA), IgG1 and IgG2a levels using rOmp16 as capture protein via indirect ELISA (ISO-2; Sigma) as described before (29). IgG1 and IgG2a were assessed at a single dilution of 1:100 in sera collected at day 30. 

Ten days after last immunization dose, splenectomy was performed and spleen lymphocytes from sacrificed mice were cultured in 24 and 96 well-plates at 4 × 10^6^ and 2.5 × 10^5^ cells per-well, respectively, using RPMI (Gibco^®^, France) supplemented with 10% FCS (Gibco^®^), Pen-Strep (Gibco^®^) and non-essential amino acids (Gibco^®^). Cultures were separately stimulated with 5 µg/ml of rOmp16 and incubated for 72 hr at 37 ^°^C and 5% of CO_2_. Lymphocytic proliferation was assayed in 96-well plates by BrdU (Roche). Cell-free culture media, collected from 24-well plates were examined by specific ELISA for evaluation of IL-4 and IFN-γ levels (R&D).

***Protective immunity assessment***

Four weeks after the last protein booster dose, 8 mice from each group were challenged intraperitoneally with 10^5^ CFU of *B. abortus* 544 and 8 mice were challenged similarly infected with *B. melitensis* 16M pathogenic strains. Mice were maintained under strict biosafety considerations for 14 days. Then, mice were sacrificed, their spleen were removed and homogenized, and dilutions from spleen suspensions were inoculated and spread on Brucella agar plates (duplicates for each mouse). Plates were incubated at 35 ^°^C for 72 hr; *Brucella* colonies were counted subsequently.

***Statistical analysis***

Data from all experiments were collected in and analyzed with the software IBM® SPSS® Statistics (Version 22.0) using the one-way analysis of variance (ANOVA) and differences between groups were processed with LSD test or Games-Howell test for unequal variances upon homogeneity status.

## Results

***Molecular cloning, protein expression, and purification***

*Omp16* gene was successfully cloned in pJET1.2 and subsequently sub cloned in pET-28a (+). Alignment of recombinant pJET-*Omp16* and pET23-*Omp16* sequencing results with reference sequences showed complete identity and no mutation in cloned ORF.

**Figure 1:**
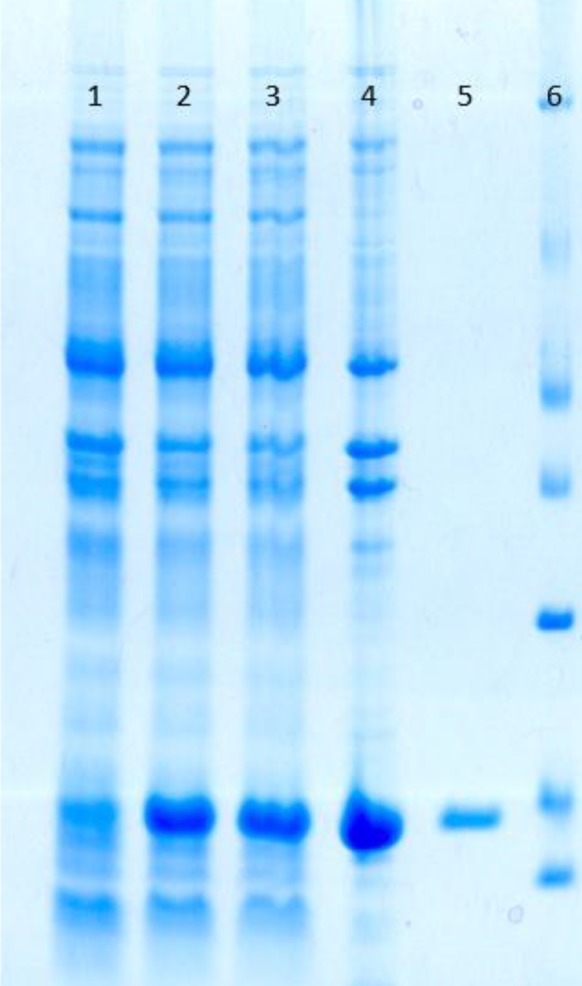
Omp16 gene expression was analyzed in SDS-PAGE. Moreover, the efficiency of purification process of rOMP16 was showed in lane 5. Recombinant host was induced with IPTG in order to express rOMP16 during 4 hr

SDS-PAGE analysis of induced culture samples of *E. coli *BL21(DE3) which was transformed with pET23-*Omp16* compared to non-induced one and non-transformed host shows the expression of an approximately ~16kDa protein (Figure 1). The expression reaches the maximum rate 2 hr after induction and remains unchanged in next hour. Purification of the protein was fulfilled using Ni-NTA resin (Qiagen; USA) via a hybrid method of denaturation and renaturation (Figure 1). A significant high yield of the rOmp16 was achieved through this method, which was measured to 3.4 mg/1 lit of induced culture.

***Western blot analysis***

**Figure 2:**
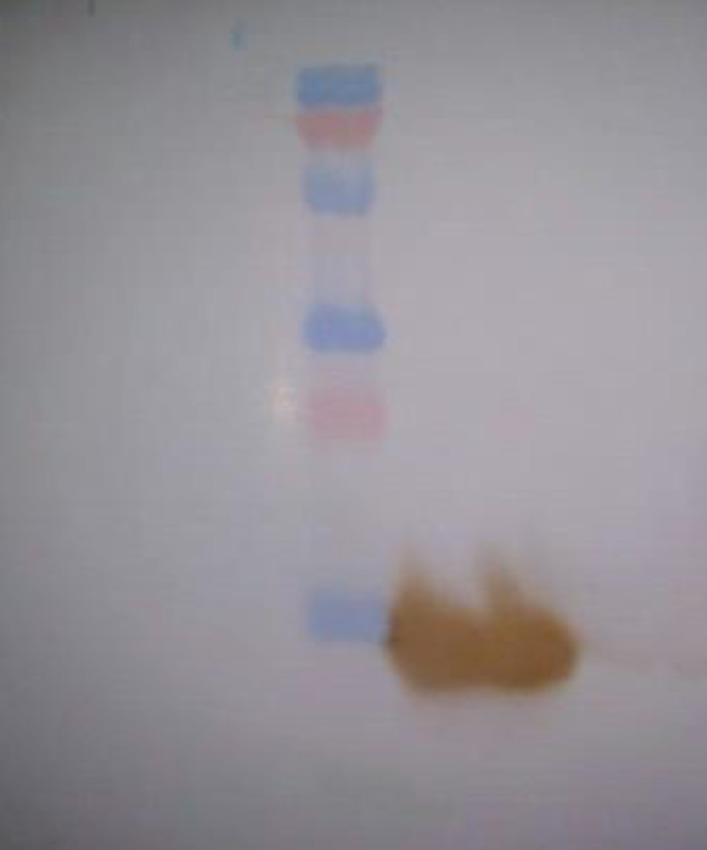
The Western blot detection of purified rOmp16

Purified recombinant rOmp16 was successfully recognized by rabbit polyclonal anti-serum and sera from three microbiologically confirmed hospitalized patients (Figure 2). This indicates that epitopes of rOmp16 are at least partially similar to the native component.

***Immunoglobulin G response***

**Figure 3:**
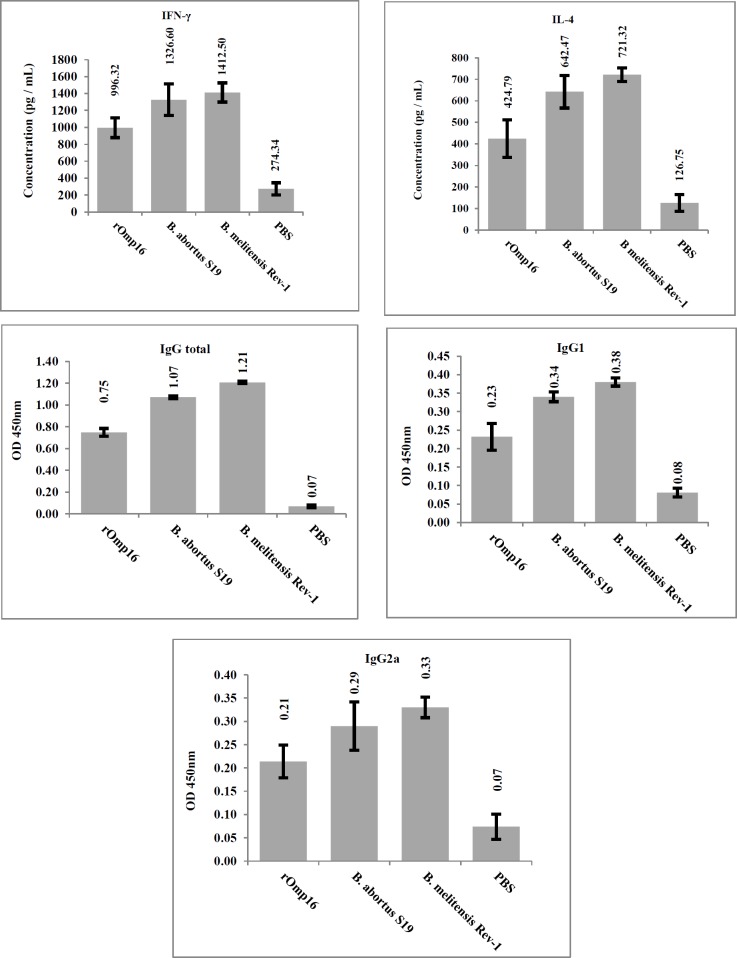
The immunoassay test results were showed mice which immunes by rOmp16 has significant difference with non-immune group. Moreover, there are no significant difference among third groups (except PBS group)

Levels of total serum IgG total, IgG1 and IgG2a subclasses are illustrated (Figure 3). Level of all antigen-specific IgG subclasses was high in mice immunized with rOmp16 (*P*<0.05) in comparison with PBS group. 

***Lymphocyte response***

Lymphocyte stimulation indices and cytokine levels produced from lymphocyte cultures showed that mice which were immunized with rOmp16 produced high levels of IFN-γ, and IL-4 in response to stimulation with the same protein (*P*≤0.05) (Figure 3). 

***Protective immunity***

Mice were challenged intraperitoneally with *B. abortus* 544 and *B. melitensis* 16M and showed the best protection as it was expected (*P*<0.05). Mice which were immunized by rOmp16 also showed a significant protection against both pathogenic strains (*P*<0.05). Protection conferred by rOmp16 was not statistically different between mice which were vaccinated with *B. abortus* S19 and *B. melitensis *Rev-1 (*P*≥0.05).

***Colony count of splenocytes***

**Figure 4:**
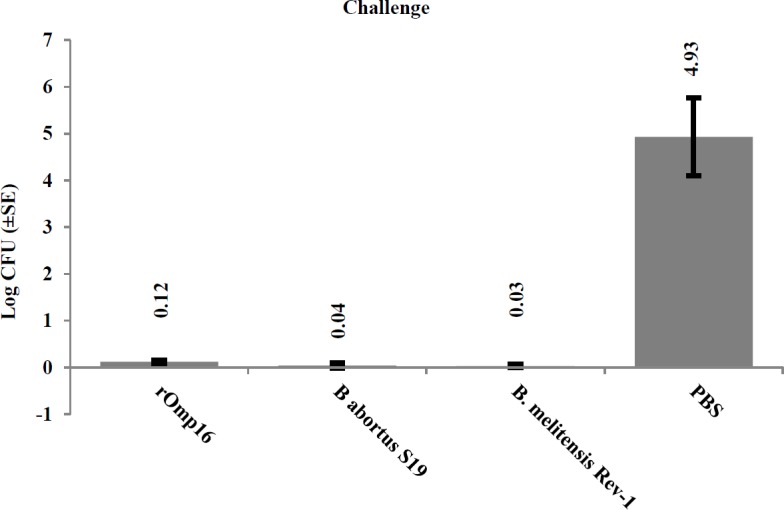
Colony count of splenocye related to each groups Colony count of Brucell spp. in different groups of Mice spleen culture on brucella agar plates

At post-infection, mice were euthanized and spleen was removed. Figure 4 shows a chart which colony count of every groups. There should be a clear reduction in the number of colonies as CFU. If the mouse has cleared the *Brucella* infection (i.e. detection limit = 100 CFU/tissue), then <5 *Brucella *colonies will be present on the brucella agar plate. 

Columns 1, 2, 3 and 4 related to the before and two, three and four hours after the induction of expression the rOmp16 by the IPTG, respectively. Column 5 is purified rOmp16 and column 6 is protein marker (down to up respectively, 14.4, 18.4, 25, 35, 45, 66.2 and 116 KDa)

Prestained protein marker contain 20, 30, 35, 45, 50, 55 and 60 KDa

## Discussion

Brucellosis is a globally spread zoonotic infection and is known to be endemic in most regions of the developing countries (31-38). The main strategy for controlling the disease is vaccination the livestock and elimination the infected animals; there are no confirmed vaccines for human (15, 33, 39-41). *Brucella *proteins, especially those located in the outer membrane of the bacterial cell interact with host immune system upon infection and may serve as effective candidates for subunit vaccines (16, 42-45). Although the major antigen is lipopolysaccharide of smooth *Brucella*, outer membrane proteins are important in host immune responses to the pathogen (14, 46). Omp16 as a major outer surface antigens of *Brucella* is a porin protein, which has been previously shown that its antibody was recognized in serum of brucellosis patients (47-50). These properties suggest that Omp16 can be investigated as a candidate for subunit vaccines against Brucellosis.

Omp16 was successfully produced and purified in *E. coli* bacteria with a significant purity and yield. In our strategy, we used pET23a (+) as expression vector which adds a minimum of amino acids to the resulting protein. Sung *et al.* reported the production of Omp16 in a pMAL system in which the target is inserted down-stream of *the male *gene which encodes maltose-binding protein (MBP) as compared with current study (19). The target protein is fused with the MBP as a solubility tag and purification system which is more different from the native one. Our method for purification the rOmp16, yielded a significant amount of soluble protein to which no additional solubility tags are attached and is more closely related to the native protein structure. Purified rOmp16 was recognized by rabbit polyclonal anti serum against *Brucella* outer membrane protein complex which suggest that the produced protein has antigenic properties to the native bacterial OMP. Reaction with antibodies in sera from brucellosis patients implies that specific immune responses are developed against Omp16 upon infection of the host with *Brucella*. 

Total IgG response to the rOmp16 was significant and indicated that the protein is a potent antigen which can elicit the remarkable antibody response in immunized mice. The specific IgG1 and IgG2a levels also show that the protein is capable to inducing both subclasses with a significant difference rather than the non-immunized mice.

Pasquerich *et al.* in Argentina have been established that immunization with purified lipidated Omp19 in adjuvant elicits protection against *B.* *abortus *infection. They are also founded that Omp16 of *B. abortus* as a recombinant lipoprotein in adjuvant elicited protection against *B. abortus *infection, further confirming that Omp16 is an important candidate for a vaccine against *Brucella*. They are showed that immunization with lipidated Omp16 (L-Omp16) or L-Omp19 in incomplete Freund’s adjuvant (IFA) conferred significant protection against *B. abortus *infection and vaccination with unlipidated Omp16 (U-Omp16) or U-Omp19 in IFA induced a higher degree of protection than the respective lipidated versions. Overall the results of these researchers indicate that U-Omp16 or U-Omp19 would be a useful candidate for a subunit vaccine against human and animal brucellosis (25).

Lymphocyte proliferation responses and high level of IFN-γ production in splenocyte cultures from mice which were immunized with rOmp16 clearly show the development of the Th-1 type of immune responses against rOmp16. Although it is less than attenuated vaccine strains, immunization with rOmp16 elicited significant protection in BALB/c mice against both *B. abortus* 544 and *B. melitensis *16M pathogenic strais which is in full consistency with cytokine responses. The low levels of antibodies or cytokines in mice which received attenuated vaccines may be attributable to the lower amount of Omp16 in the bacterial cell as compared to pure protein dose; this may result in the development of the smaller number of specific T-lymphocyte clones which recognize Omp16 epitopes. 

## Conclusion

Put together, rOmp16 is capable of inducing both antibody and cytokine responses which indicate a Th-1 type response and suggest Omp16 as an outstanding candidate for designing subunit vaccines against brucellosis. 
